# Poly[μ_3_-aqua-aqua(μ_3_-3,5-dinitro­benzoato-κ*O* ^1^:*O* ^3^:*O* ^5^)caesium]

**DOI:** 10.1107/S1600536812037130

**Published:** 2012-09-05

**Authors:** Graham Smith

**Affiliations:** a Science and Engineering Faculty, Queensland University of Technology, GPO Box 2434, Brisbane, Queensland 4001, Australia

## Abstract

In the structure of the title complex, [Cs(C_7_H_3_N_2_O_6_)(H_2_O)_2_]_*n*_, the Cs salt of 3,5-dinitro­benzoic acid, the metal complex centres have have irregular CsO_8_ coordination, comprising two water mol­ecules (one triply bridging and the other monodentate) and four O-atom donors from two nitro groups and one bridging carboxyl­ate O-atom donor from the ligand. Intra-unit O—H⋯O hydrogen-bonding inter­actions involving both water mol­ecules are observed in the three-dimensional polymeric complex structure.

## Related literature

For exanples of structures of alkali metal complexes with 3,5-dinitro­benzoic acid, see: Yang & Ng (2007 ▶) (Li, Na); Tiekink *et al.* (1990 ▶); Jones *et al.* (2005 ▶); Madej *et al.* (2007 ▶) (Na); Miao & Fan (2011 ▶); Miao *et al.* (2011 ▶) (Rb). For examples of Cs complexes with nitro­benzoic acids, see: Smith & Wermuth (2011*a* ▶,*b* ▶).
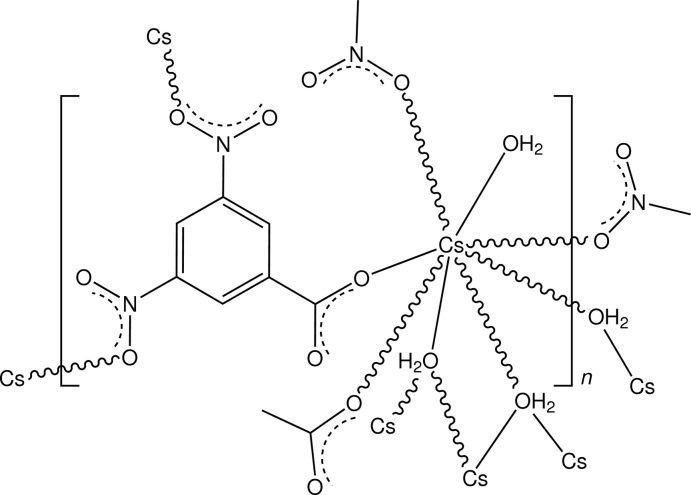


## Experimental

### 

#### Crystal data

[Cs(C_7_H_3_N_2_O_6_)(H_2_O)_2_]
*M* *_r_* = 380.06
Monoclinic, 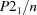

*a* = 15.1249 (5) Å
*b* = 4.6223 (1) Å
*c* = 17.1024 (6) Å
β = 107.782 (4)°
*V* = 1138.54 (7) Å^3^
*Z* = 4
Mo *K*α radiation
μ = 3.29 mm^−1^
*T* = 200 K
0.28 × 0.15 × 0.06 mm

#### Data collection

Oxford Diffraction Gemini-S CCD-detector diffractometer
Absorption correction: multi-scan (*CrysAlis PRO*; Agilent, 2012 ▶) *T*_min_ = 0.792, *T*_max_ = 0.980
7596 measured reflections
2652 independent reflections
2336 reflections with *I* > 2σ(*I*)
*R*_int_ = 0.030

#### Refinement

*R*[*F* ^2^ > 2σ(*F* ^2^)] = 0.024
*wR*(*F* ^2^) = 0.048
*S* = 1.05
2652 reflections
179 parameters
H atoms treated by a mixture of independent and constrained refinement
Δρ_max_ = 0.47 e Å^−3^
Δρ_min_ = −0.56 e Å^−3^

### 

Data collection: *CrysAlis PRO* (Agilent, 2012 ▶); cell refinement: *CrysAlis PRO*; data reduction: *CrysAlis PRO*; program(s) used to solve structure: *SIR92* (Altomare *et al.*, 1993 ▶); program(s) used to refine structure: *SHELXL97* (Sheldrick, 2008 ▶) within *WinGX* (Farrugia, 1999 ▶); molecular graphics: *PLATON* (Spek, 2009 ▶); software used to prepare material for publication: *PLATON*.

## Supplementary Material

Crystal structure: contains datablock(s) global, I. DOI: 10.1107/S1600536812037130/ng5289sup1.cif

Structure factors: contains datablock(s) I. DOI: 10.1107/S1600536812037130/ng5289Isup2.hkl

Supplementary material file. DOI: 10.1107/S1600536812037130/ng5289Isup3.cml

Additional supplementary materials:  crystallographic information; 3D view; checkCIF report

## Figures and Tables

**Table 1:** Selected bond lengths (Å)

Cs1—O1*W*	3.087 (2)
Cs1—O2*W*	3.282 (2)
Cs1—O12	3.1751 (16)
Cs1—O12^i^	3.1120 (17)
Cs1—O1*W* ^ii^	3.261 (2)
Cs1—O32^iii^	3.244 (2)
Cs1—O1*W* ^iv^	3.346 (2)
Cs1—O52^v^	3.271 (2)

Symmetry codes: (i) 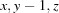; (ii) 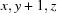; (iii) 
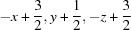
; (iv) 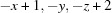; (v) 
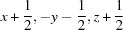
.

**Table 2:** Hydrogen-bond geometry (Å, °)

*D*—H⋯*A*	*D*—H	H⋯*A*	*D*⋯*A*	*D*—H⋯*A*
O1*W*—H11*W*⋯O2*W*	0.80 (4)	1.98 (4)	2.734 (3)	159 (3)
O1*W*—H12*W*⋯O12^iv^	0.83 (4)	2.25 (4)	3.016 (3)	155 (4)
O2*W*—H21*W*⋯O11^vi^	0.83 (4)	1.94 (4)	2.764 (3)	174 (3)
O2*W*—H22*W*⋯O11^vii^	0.85 (4)	1.96 (4)	2.797 (3)	168 (3)

Symmetry codes: (iv) 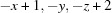; (vi) 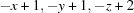; (vii) 
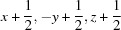
.

**Fig. 1.**
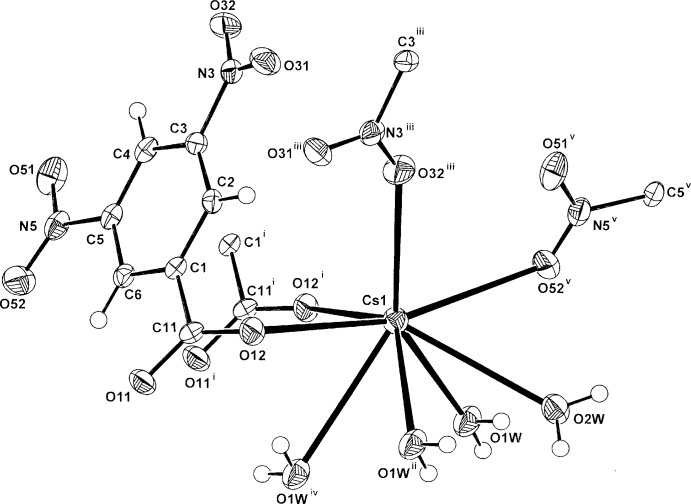
The molecular configuration and atom-numbering scheme for the title compound, with non-H atoms drawn as 50% probability ellipsoids. For symmetry codes: see Table 1.

**Fig. 2.**
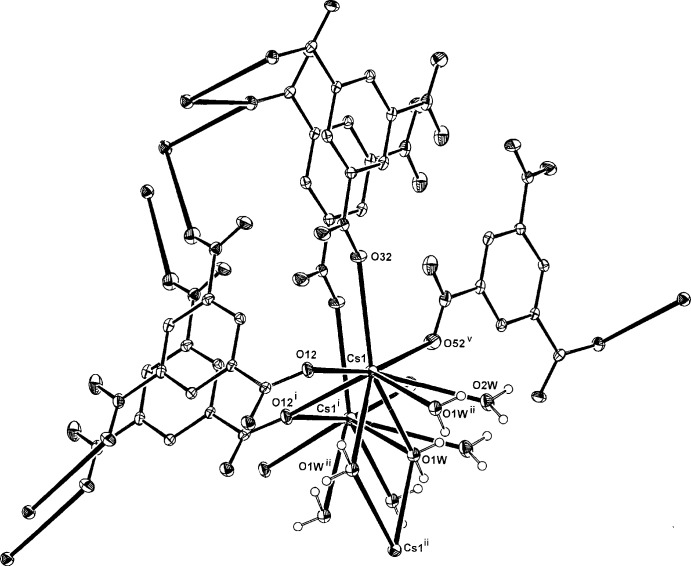
A section of the three-dimensional coordination polymer showing inter-unit Cs···Cs associations and 30% probability ellipsoids.

**Fig. 3.**
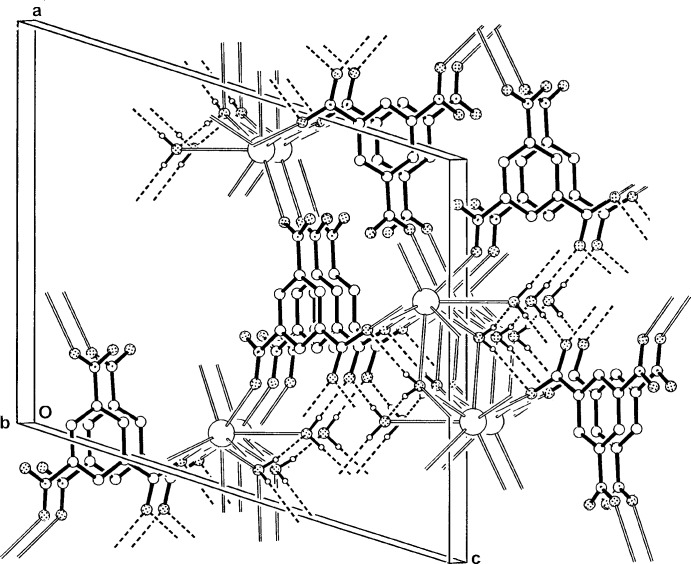
The packing in the unit cell viewed down the the b axial direction showing intra-unit hydrogen-bonding associations as dashed lines. Non-interactive H atoms are omitted. For symmetry codes, see Fig. 1 and Table 2.

## supplementary crystallographic information

### Comment

3,5-Dinitrobenzoic acid (DNBA) has been a popular ligand used alone or in mixed-ligand applications for metal complexation and the structures of a large number of its complexes have been reported. With the alkali metals the structures of the complex salts with Li and Na (Yang & Ng, 2007; Jones *et al.*, 2005): the complex salt adduct with Na (Tiekink *et al.*, 1990; Madej *et al.*, 2007) and the Rb complex salt and salt adduct (Miao & Fan, 2011; Miao *et al.*, 2011) are known but the structure of the Cs complex salt has not been reported. The reaction of 3,5-dinitrobenzoic acid with caesium hydroxide in aqueous ethanol gave crystals of the title compound [Cs(C_7_H_3_N_2_O_6_)(H_2_O)_2_]_n_ and the structure is reported here. In the Rb and Cs complexes with the nitro-substituted aromatic carboxylic acids, expanded metal coordination spheres together with polymeric structures are common, in which ligands are bridging, *e.g.* anhydrous rubidium 3,5-dinitrobenzoate (8-coordinate) (Miao & Fan, 2011); tetracaesium bis(5-nitroisophthalate) heptahydrate (6- and 8-coordinate) (Smith & Wermuth, 2011*a*) and caesium bis(2-nitroanthranilate) dihydrate (7- and 9-coordinate) (Smith & Wermuth, 2011*b*).

In the structure of the title compound the CsO_8_ complex unit (Fig. 1) is irregular 8-coordinate [Cs—O range, 3.087 (2)–3.346 (2) Å] (Table 1), comprising two water molecules (one triply bridging, the other monodentate) and four O-donors from two nitro groups and one bridging carboxyl-O donor group from the ligand. In the three-dimensional polymeric complex structure (Fig. 2), the rings of the DNBA ligands layer down the short *b* axis of the unit cell with a ring centroid separation of 4.6223 (1) Å (the *b* cell dimension) (Fig. 3). Present also are intra-polymer O—H···O hydrogen-bonding interactions involving both water molecules (Table 2).

### Experimental

The title compound was synthesized by heating together under reflux for 10 minutes, 0.5 mmol of caesium hydroxide and 0.5 mmol of 3,5-dinitrobenzoic acid in 20 ml of 10% ethanol–water. Room temperature evaporation of the solution to incipient dryness gave yellow needle crystals of the title complex from which a specimen was cleaved for the X-ray analysis.

### Refinement

Hydrogen atoms of the coordinated water molecules were located in a difference-Fourier synthesis and both positional and isotropic displacement parameters were allowed to refine. Other H-atoms were included at calculated positions and were allowed to ride, with C—H = 0.93 Å and *U*_iso_(H) = 1.2*U*_eq_(C).

### Crystal data

**Table:**

[Cs(C_7_H_3_N_2_O_6_)(H_2_O)_2_]	*F*(000) = 728
*M**_r_* = 380.06	*D*_x_ = 2.217 Mg m^−^^3^
Monoclinic, *P*2_1_/*n*	Mo *K*α radiation, λ = 0.71073 Å
Hall symbol: -P 2yn	Cell parameters from 3275 reflections
*a* = 15.1249 (5) Å	θ = 3.2–28.8°
*b* = 4.6223 (1) Å	µ = 3.29 mm^−^^1^
*c* = 17.1024 (6) Å	*T* = 200 K
β = 107.782 (4)°	Needle, yellow
*V* = 1138.54 (7) Å^3^	0.28 × 0.15 × 0.06 mm
*Z* = 4	

### Data collection

**Table:**

Oxford Diffraction Gemini-S CCD-detector diffractometer	2652 independent reflections
Radiation source: Enhance (Mo) X-ray source	2336 reflections with *I* > 2σ(*I*)
Graphite monochromator	*R*_int_ = 0.030
Detector resolution: 16.077 pixels mm^-1^	θ_max_ = 28.9°, θ_min_ = 3.2°
ω scans	*h* = −20→20
Absorption correction: multi-scan (*CrysAlis PRO*; Agilent, 2012)	*k* = −6→5
*T*_min_ = 0.792, *T*_max_ = 0.980	*l* = −22→22
7596 measured reflections	

### Refinement

**Table:**

Refinement on *F*^2^	Primary atom site location: structure-invariant direct methods
Least-squares matrix: full	Secondary atom site location: difference Fourier map
*R*[*F*^2^ > 2σ(*F*^2^)] = 0.024	Hydrogen site location: inferred from neighbouring sites
*wR*(*F*^2^) = 0.048	H atoms treated by a mixture of independent and constrained refinement
*S* = 1.05	*w* = 1/[σ^2^(*F*_o_^2^) + (0.0172*P*)^2^] where *P* = (*F*_o_^2^ + 2*F*_c_^2^)/3
2652 reflections	(Δ/σ)_max_ = 0.001
179 parameters	Δρ_max_ = 0.47 e Å^−^^3^
0 restraints	Δρ_min_ = −0.56 e Å^−^^3^

### Special details

**Table:**

Geometry. Bond distances, angles *etc*. have been calculated using the rounded fractional coordinates. All su's are estimated from the variances of the (full) variance-covariance matrix. The cell e.s.d.'s are taken into account in the estimation of distances, angles and torsion angles
Refinement. Refinement of *F*^2^ against ALL reflections. The weighted *R*-factor *wR* and goodness of fit *S* are based on *F*^2^, conventional *R*-factors *R* are based on *F*, with *F* set to zero for negative *F*^2^. The threshold expression of *F*^2^ > σ(*F*^2^) is used only for calculating *R*-factors(gt) *etc*. and is not relevant to the choice of reflections for refinement. *R*-factors based on *F*^2^ are statistically about twice as large as those based on *F*, and *R*- factors based on ALL data will be even larger.

### Fractional atomic coordinates and isotropic or equivalent isotropic displacement parameters (Å^2^)

**Table:**

	*x*	*y*	*z*	*U*_iso_*/*U*_eq_	
Cs1	0.63127 (1)	0.11431 (3)	0.94980 (1)	0.0232 (1)	
O1W	0.57824 (16)	−0.3598 (5)	1.05448 (13)	0.0315 (7)	
O2W	0.69998 (16)	0.0306 (5)	1.14991 (13)	0.0311 (7)	
O11	0.37491 (13)	0.6190 (4)	0.75558 (11)	0.0278 (6)	
O12	0.52278 (14)	0.6236 (3)	0.83580 (11)	0.0266 (6)	
O31	0.74660 (14)	0.3113 (4)	0.68972 (12)	0.0315 (6)	
O32	0.71260 (15)	−0.0756 (4)	0.61600 (12)	0.0351 (7)	
O51	0.39421 (16)	−0.3890 (4)	0.51426 (12)	0.0351 (7)	
O52	0.28880 (15)	−0.1482 (4)	0.54804 (12)	0.0381 (7)	
N3	0.69218 (16)	0.1222 (4)	0.65482 (13)	0.0234 (7)	
N5	0.36953 (17)	−0.2001 (5)	0.55368 (12)	0.0246 (7)	
C1	0.48439 (18)	0.3386 (5)	0.71545 (14)	0.0169 (7)	
C2	0.57543 (18)	0.3203 (5)	0.71383 (14)	0.0177 (7)	
C3	0.59685 (18)	0.1317 (5)	0.65956 (15)	0.0186 (7)	
C4	0.53156 (19)	−0.0458 (5)	0.60715 (14)	0.0191 (7)	
C5	0.44224 (19)	−0.0198 (5)	0.60976 (14)	0.0187 (7)	
C6	0.41608 (18)	0.1685 (5)	0.66192 (14)	0.0187 (7)	
C11	0.45850 (19)	0.5455 (5)	0.77413 (15)	0.0186 (7)	
H2	0.62150	0.43370	0.74890	0.0210*	
H4	0.54740	−0.17570	0.57210	0.0230*	
H6	0.35450	0.18100	0.66120	0.0220*	
H11W	0.616 (3)	−0.280 (7)	1.091 (2)	0.038 (10)*	
H12W	0.559 (3)	−0.478 (7)	1.081 (2)	0.055 (12)*	
H21W	0.677 (3)	0.124 (6)	1.180 (2)	0.052 (12)*	
H22W	0.750 (3)	−0.041 (6)	1.1818 (19)	0.037 (9)*	

### Atomic displacement parameters (Å^2^)

**Table:**

	*U*^11^	*U*^22^	*U*^33^	*U*^12^	*U*^13^	*U*^23^
Cs1	0.0226 (1)	0.0231 (1)	0.0235 (1)	−0.0002 (1)	0.0063 (1)	0.0030 (1)
O1W	0.0308 (13)	0.0405 (12)	0.0214 (10)	−0.0117 (10)	0.0053 (9)	0.0000 (10)
O2W	0.0259 (12)	0.0355 (11)	0.0284 (11)	0.0046 (10)	0.0032 (10)	−0.0094 (10)
O11	0.0174 (10)	0.0373 (11)	0.0279 (10)	0.0052 (8)	0.0059 (8)	−0.0072 (8)
O12	0.0229 (11)	0.0330 (10)	0.0210 (9)	−0.0005 (8)	0.0023 (8)	−0.0092 (8)
O31	0.0204 (11)	0.0385 (11)	0.0356 (11)	−0.0040 (9)	0.0084 (9)	−0.0042 (9)
O32	0.0310 (12)	0.0442 (12)	0.0324 (11)	0.0130 (9)	0.0130 (10)	−0.0079 (9)
O51	0.0492 (15)	0.0248 (10)	0.0266 (10)	−0.0040 (9)	0.0045 (10)	−0.0093 (8)
O52	0.0252 (12)	0.0548 (13)	0.0342 (11)	−0.0143 (10)	0.0089 (9)	−0.0132 (10)
N3	0.0223 (13)	0.0294 (12)	0.0180 (11)	0.0075 (10)	0.0055 (9)	0.0042 (9)
N5	0.0304 (15)	0.0246 (11)	0.0169 (11)	−0.0075 (10)	0.0044 (10)	−0.0012 (9)
C1	0.0203 (14)	0.0179 (11)	0.0128 (11)	−0.0002 (10)	0.0055 (10)	0.0013 (9)
C2	0.0171 (13)	0.0182 (11)	0.0161 (12)	−0.0004 (10)	0.0024 (10)	0.0025 (10)
C3	0.0188 (14)	0.0198 (12)	0.0178 (12)	0.0039 (10)	0.0067 (10)	0.0057 (10)
C4	0.0278 (15)	0.0153 (11)	0.0141 (12)	0.0013 (10)	0.0063 (10)	−0.0001 (9)
C5	0.0244 (15)	0.0175 (11)	0.0130 (11)	−0.0039 (11)	0.0039 (10)	−0.0005 (10)
C6	0.0197 (14)	0.0207 (12)	0.0152 (11)	−0.0023 (10)	0.0048 (10)	0.0034 (10)
C11	0.0220 (14)	0.0192 (12)	0.0168 (12)	−0.0014 (10)	0.0091 (10)	0.0022 (10)

### Geometric parameters (Å, º)

**Table:**

Cs1—O1W	3.087 (2)	O1W—H11W	0.80 (4)
Cs1—O2W	3.282 (2)	O2W—H21W	0.83 (4)
Cs1—O12	3.1751 (16)	O2W—H22W	0.85 (4)
Cs1—O12^i^	3.1120 (17)	N3—C3	1.469 (4)
Cs1—O1W^ii^	3.261 (2)	N5—C5	1.476 (3)
Cs1—O32^iii^	3.244 (2)	C1—C2	1.388 (4)
Cs1—O1W^iv^	3.346 (2)	C1—C6	1.395 (3)
Cs1—O52^v^	3.271 (2)	C1—C11	1.522 (3)
O11—C11	1.253 (4)	C2—C3	1.382 (3)
O12—C11	1.250 (3)	C3—C4	1.382 (4)
O31—N3	1.224 (3)	C4—C5	1.371 (4)
O32—N3	1.224 (3)	C5—C6	1.388 (3)
O51—N5	1.229 (3)	C2—H2	0.9300
O52—N5	1.219 (4)	C4—H4	0.9300
O1W—H12W	0.82 (4)	C6—H6	0.9300
			
O1W—Cs1—O2W	50.74 (6)	H11W—O1W—H12W	100 (4)
O1W—Cs1—O12	134.87 (6)	Cs1^i^—O1W—H11W	123 (3)
O1W—Cs1—O12^i^	70.44 (5)	Cs1^iv^—O1W—H11W	108 (3)
O1W—Cs1—O1W^ii^	93.43 (6)	Cs1^i^—O1W—H12W	90 (3)
O1W—Cs1—O32^iii^	149.39 (6)	Cs1^iv^—O1W—H12W	75 (3)
O1W—Cs1—O1W^iv^	80.87 (6)	Cs1—O1W—H11W	83 (2)
O1W—Cs1—O52^v^	60.79 (6)	H21W—O2W—H22W	105 (3)
O2W—Cs1—O12	131.87 (5)	Cs1—O2W—H22W	131 (2)
O2W—Cs1—O12^i^	120.39 (5)	Cs1—O2W—H21W	122 (2)
O1W^ii^—Cs1—O2W	64.41 (6)	O32—N3—C3	117.8 (2)
O2W—Cs1—O32^iii^	112.44 (6)	O31—N3—C3	118.5 (2)
O1W^iv^—Cs1—O2W	93.38 (6)	O31—N3—O32	123.7 (3)
O2W—Cs1—O52^v^	55.78 (6)	O51—N5—O52	124.1 (2)
O12—Cs1—O12^i^	94.64 (4)	O51—N5—C5	117.8 (2)
O1W^ii^—Cs1—O12	67.47 (5)	O52—N5—C5	118.0 (2)
O12—Cs1—O32^iii^	75.75 (5)	C6—C1—C11	119.9 (2)
O1W^iv^—Cs1—O12	55.02 (5)	C2—C1—C6	119.5 (2)
O12—Cs1—O52^v^	164.29 (6)	C2—C1—C11	120.5 (2)
O1W^ii^—Cs1—O12^i^	135.75 (6)	C1—C2—C3	119.3 (2)
O12^i^—Cs1—O32^iii^	113.90 (5)	C2—C3—C4	122.8 (3)
O1W^iv^—Cs1—O12^i^	85.35 (5)	N3—C3—C2	119.4 (2)
O12^i^—Cs1—O52^v^	90.21 (5)	N3—C3—C4	117.7 (2)
O1W^ii^—Cs1—O32^iii^	100.85 (5)	C3—C4—C5	116.3 (2)
O1W^ii^—Cs1—O1W^iv^	50.88 (6)	C4—C5—C6	123.6 (2)
O1W^ii^—Cs1—O52^v^	118.15 (5)	N5—C5—C6	118.0 (3)
O1W^iv^—Cs1—O32^iii^	128.87 (5)	N5—C5—C4	118.4 (2)
O32^iii^—Cs1—O52^v^	88.62 (5)	C1—C6—C5	118.4 (3)
O1W^iv^—Cs1—O52^v^	140.47 (5)	O11—C11—O12	126.9 (2)
Cs1—O1W—Cs1^i^	93.43 (6)	O11—C11—C1	116.5 (2)
Cs1—O1W—Cs1^iv^	99.13 (6)	O12—C11—C1	116.6 (2)
Cs1^i^—O1W—Cs1^iv^	129.12 (7)	C1—C2—H2	120.00
Cs1—O12—C11	115.28 (13)	C3—C2—H2	120.00
Cs1—O12—Cs1^ii^	94.64 (5)	C3—C4—H4	122.00
Cs1^ii^—O12—C11	149.86 (14)	C5—C4—H4	122.00
Cs1^vi^—O32—N3	148.16 (18)	C1—C6—H6	121.00
Cs1^vii^—O52—N5	117.78 (15)	C5—C6—H6	121.00
Cs1—O1W—H12W	174 (3)		
			
O2W—Cs1—O1W—Cs1^i^	−127.44 (9)	O1W—Cs1—O1W^iv^—Cs1^iv^	0.00 (6)
O2W—Cs1—O1W—Cs1^iv^	102.04 (8)	O2W—Cs1—O1W^iv^—Cs1^iv^	−49.33 (6)
O12—Cs1—O1W—Cs1^i^	118.81 (6)	O12—Cs1—O1W^iv^—Cs1^iv^	169.89 (8)
O12—Cs1—O1W—Cs1^iv^	−11.71 (9)	O1W—Cs1—O52^v^—N5^v^	−167.08 (18)
O12^i^—Cs1—O1W—Cs1^i^	42.25 (5)	O2W—Cs1—O52^v^—N5^v^	−106.86 (18)
O12^i^—Cs1—O1W—Cs1^iv^	−88.27 (6)	Cs1—O12—C11—O11	122.9 (2)
O1W^ii^—Cs1—O1W—Cs1^i^	179.98 (9)	Cs1—O12—C11—C1	−57.0 (3)
O1W^ii^—Cs1—O1W—Cs1^iv^	49.48 (6)	Cs1^ii^—O12—C11—O11	−49.7 (5)
O32^iii^—Cs1—O1W—Cs1^i^	−61.86 (12)	Cs1^ii^—O12—C11—C1	130.5 (3)
O32^iii^—Cs1—O1W—Cs1^iv^	167.61 (7)	Cs1^vi^—O32—N3—O31	−0.6 (4)
O1W^iv^—Cs1—O1W—Cs1^i^	130.53 (6)	Cs1^vi^—O32—N3—C3	179.48 (19)
O1W^iv^—Cs1—O1W—Cs1^iv^	−0.02 (12)	Cs1^vii^—O52—N5—O51	12.2 (3)
O52^v^—Cs1—O1W—Cs1^i^	−59.47 (6)	Cs1^vii^—O52—N5—C5	−169.48 (15)
O52^v^—Cs1—O1W—Cs1^iv^	170.01 (8)	O31—N3—C3—C2	−11.0 (3)
O1W—Cs1—O12—C11	−63.1 (2)	O31—N3—C3—C4	167.3 (2)
O1W—Cs1—O12—Cs1^ii^	113.15 (7)	O32—N3—C3—C2	169.0 (2)
O2W—Cs1—O12—C11	−135.23 (18)	O32—N3—C3—C4	−12.7 (3)
O2W—Cs1—O12—Cs1^ii^	41.03 (9)	O51—N5—C5—C4	8.4 (3)
O12^i^—Cs1—O12—C11	3.7 (2)	O51—N5—C5—C6	−172.0 (2)
O12^i^—Cs1—O12—Cs1^ii^	−180.00 (7)	O52—N5—C5—C4	−170.0 (2)
O1W^ii^—Cs1—O12—C11	−134.4 (2)	O52—N5—C5—C6	9.6 (3)
O1W^ii^—Cs1—O12—Cs1^ii^	41.90 (6)	C6—C1—C2—C3	−0.5 (3)
O32^iii^—Cs1—O12—C11	117.2 (2)	C11—C1—C2—C3	−179.6 (2)
O32^iii^—Cs1—O12—Cs1^ii^	−66.50 (5)	C2—C1—C6—C5	1.5 (3)
O1W^iv^—Cs1—O12—C11	−77.3 (2)	C11—C1—C6—C5	−179.4 (2)
O1W^iv^—Cs1—O12—Cs1^ii^	98.99 (7)	C2—C1—C11—O11	158.1 (2)
O1W—Cs1—O12^i^—Cs1^i^	−43.76 (6)	C2—C1—C11—O12	−22.1 (3)
O1W—Cs1—O12^i^—C11^i^	129.5 (4)	C6—C1—C11—O11	−21.0 (3)
O2W—Cs1—O12^i^—Cs1^i^	−34.52 (8)	C6—C1—C11—O12	158.9 (2)
O2W—Cs1—O12^i^—C11^i^	138.7 (4)	C1—C2—C3—N3	177.0 (2)
O12—Cs1—O12^i^—Cs1^i^	180.00 (7)	C1—C2—C3—C4	−1.2 (4)
O12—Cs1—O12^i^—C11^i^	−6.8 (4)	N3—C3—C4—C5	−176.4 (2)
O1W—Cs1—O1W^ii^—Cs1^ii^	179.98 (9)	C2—C3—C4—C5	1.9 (4)
O2W—Cs1—O1W^ii^—Cs1^ii^	137.03 (8)	C3—C4—C5—N5	178.7 (2)
O12—Cs1—O1W^ii^—Cs1^ii^	−42.25 (5)	C3—C4—C5—C6	−0.9 (4)
O1W—Cs1—O32^iii^—N3^iii^	−174.4 (3)	N5—C5—C6—C1	179.7 (2)
O2W—Cs1—O32^iii^—N3^iii^	−124.7 (3)	C4—C5—C6—C1	−0.8 (4)
O12—Cs1—O32^iii^—N3^iii^	5.1 (3)		

Symmetry codes: (i) *x*, *y*−1, *z*; (ii) *x*, *y*+1, *z*; (iii) −*x*+3/2, *y*+1/2, −*z*+3/2; (iv) −*x*+1, −*y*, −*z*+2; (v) *x*+1/2, −*y*−1/2, *z*+1/2; (vi) −*x*+3/2, *y*−1/2, −*z*+3/2; (vii) *x*−1/2, −*y*−1/2, *z*−1/2.

### Hydrogen-bond geometry (Å, º)

**Table:**

*D*—H···*A*	*D*—H	H···*A*	*D*···*A*	*D*—H···*A*
O1*W*—H11*W*···O2*W*	0.80 (4)	1.98 (4)	2.734 (3)	159 (3)
O1*W*—H12*W*···O12^iv^	0.83 (4)	2.25 (4)	3.016 (3)	155 (4)
O2*W*—H21*W*···O11^viii^	0.83 (4)	1.94 (4)	2.764 (3)	174 (3)
O2*W*—H22*W*···O11^ix^	0.85 (4)	1.96 (4)	2.797 (3)	168 (3)

Symmetry codes: (iv) −*x*+1, −*y*, −*z*+2; (viii) −*x*+1, −*y*+1, −*z*+2; (ix) *x*+1/2, −*y*+1/2, *z*+1/2.

## References

[bb1] Agilent (2012). *CrysAlis PRO* Agilent Technologies Ltd, Yarnton, England.

[bb2] Altomare, A., Cascarano, G., Giacovazzo, C. & Guagliardi, A. (1993). *J. Appl. Cryst.* **26**, 343–350.

[bb3] Farrugia, L. J. (1999). *J. Appl. Cryst.* **32**, 837–838.

[bb4] Jones, H. P., Gillon, A. L. & Davey, R. J. (2005). *Acta Cryst.* E**61**, m1131–m1132.

[bb5] Madej, A., Oleksin, B. J. & Śliwiński, J. (2007). *Pol. J. Chem.* **81**, 1201–1218.

[bb6] Miao, Y. & Fan, T. (2011). *Acta Cryst.* E**67**, m1040. 10.1107/S160053681102513X PMC3212130 22090832

[bb7] Miao, Y., Zhang, X. & Liu, C. (2011). *Acta Cryst.* E**67**, m1002. 10.1107/S1600536811023026 PMC3151889 21836829

[bb8] Sheldrick, G. M. (2008). *Acta Cryst.* A**64**, 112–122. 10.1107/S0108767307043930 18156677

[bb9] Smith, G. & Wermuth, U. D. (2011*a*). *J. Chem. Crystallogr.* **41**, 688–692.

[bb10] Smith, G. & Wermuth, U. D. (2011*b*). *Acta Cryst.* E**67**, m1047–m1048. 10.1107/S1600536811026614 PMC3212135 22090837

[bb11] Spek, A. L. (2009). *Acta Cryst* D**65**, 148–155. 10.1107/S090744490804362X PMC2631630 19171970

[bb12] Tiekink, E. R. T., Hundal, M. S., Sood, G., Kapoor, P. & Poonia, N. S. (1990). *Z. Kristallogr.* **192**, 103–109.

[bb13] Yang, G. & Ng, S. W. (2007). *Cryst. Res. Technol.* **42**, 201–206.

